# Supplementary material for: Modeling glioblastoma heterogeneity as a dynamic network of cell states
Source: Mol Syst Biol. 2021 Sep 16;17(9):e10105. doi: 10.15252/msb.202010105 (PMC8444284; doi:10.15252/msb.202010105)
Supplement: Supplementary file 6 — Source Data for Figure 5 [file MSB-17-e10105-s004.zip › Figure5A_sourcedata/GSEA_3017/nosigdb_stateE.GseaPreranked.1622795031093/FELDMAN_2020_G2-M.html]

Details for gene set FELDMAN\_2020\_G2-M[GSEA]

|  || Dataset | state63017 |
| Phenotype | NoPhenotypeAvailable |
| Upregulated in class | na\_neg |
| GeneSet | FELDMAN\_2020\_G2-M |
| Enrichment Score (ES) | -0.6226381 |
| Normalized Enrichment Score (NES) | -3.5590198 |
| Nominal p-value | 0.0 |
| FDR q-value | 0.0 |
| FWER p-Value | 0.0 |
Table: GSEA Results Summary

  

Fig 1: Enrichment plot: FELDMAN\_2020\_G2-M      
 Profile of the Running ES Score & Positions of GeneSet Members on the Rank Ordered List

  

| PROBE | GENE SYMBOL | GENE\_TITLE | RANK IN GENE LIST | RANK METRIC SCORE | RUNNING ES | CORE ENRICHMENT || 1 | CTGF |  |  | 2 | 1.276 | 0.0090 | No |
| 2 | IGFBP5 |  |  | 4 | 1.015 | 0.0169 | No |
| 3 | FABP7 |  |  | 11 | 0.920 | 0.0174 | No |
| 4 | TTYH1 |  |  | 19 | 0.731 | 0.0150 | No |
| 5 | IFITM3 |  |  | 26 | 0.682 | 0.0133 | No |
| 6 | CALD1 |  |  | 41 | 0.580 | 0.0004 | No |
| 7 | SERPINE1 |  |  | 52 | 0.526 | -0.0079 | No |
| 8 | CST3 |  |  | 86 | 0.425 | -0.0471 | No |
| 9 | BEX1 |  |  | 88 | 0.419 | -0.0446 | No |
| 10 | GPM6B |  |  | 105 | 0.391 | -0.0619 | No |
| 11 | CLU |  |  | 119 | 0.364 | -0.0755 | No |
| 12 | DCBLD2 |  |  | 165 | 0.322 | -0.1313 | No |
| 13 | FILIP1L |  |  | 167 | 0.320 | -0.1296 | No |
| 14 | CALM2 |  |  | 191 | 0.303 | -0.1569 | No |
| 15 | PLIN3 |  |  | 192 | 0.301 | -0.1541 | No |
| 16 | FTH1 |  |  | 204 | 0.293 | -0.1658 | No |
| 17 | SAT1 |  |  | 206 | 0.291 | -0.1645 | No |
| 18 | MEST |  |  | 215 | 0.285 | -0.1723 | No |
| 19 | RAN |  |  | 298 | -0.257 | -0.2769 | No |
| 20 | HMGA1 |  |  | 313 | -0.261 | -0.2928 | No |
| 21 | HNRNPDL |  |  | 322 | -0.263 | -0.3008 | No |
| 22 | TUBG1 |  |  | 323 | -0.263 | -0.2984 | No |
| 23 | NUDCD2 |  |  | 330 | -0.264 | -0.3038 | No |
| 24 | PRPSAP1 |  |  | 369 | -0.273 | -0.3509 | No |
| 25 | ADA |  |  | 371 | -0.273 | -0.3497 | No |
| 26 | TMEM237 |  |  | 383 | -0.276 | -0.3615 | No |
| 27 | HNRNPA2B1 |  |  | 391 | -0.279 | -0.3681 | No |
| 28 | LSM4 |  |  | 406 | -0.284 | -0.3838 | No |
| 29 | CHCHD2 |  |  | 423 | -0.290 | -0.4020 | No |
| 30 | OIP5 |  |  | 426 | -0.291 | -0.4020 | No |
| 31 | ILF2 |  |  | 497 | -0.313 | -0.4904 | No |
| 32 | GGH |  |  | 504 | -0.314 | -0.4954 | No |
| 33 | HNRNPH3 |  |  | 507 | -0.316 | -0.4951 | No |
| 34 | KIF20A |  |  | 523 | -0.321 | -0.5118 | No |
| 35 | RHEB |  |  | 528 | -0.323 | -0.5140 | No |
| 36 | LSM3 |  |  | 530 | -0.323 | -0.5124 | No |
| 37 | GPSM2 |  |  | 550 | -0.334 | -0.5341 | No |
| 38 | FAM83D |  |  | 559 | -0.340 | -0.5415 | No |
| 39 | TROAP |  |  | 562 | -0.341 | -0.5410 | No |
| 40 | LSM5 |  |  | 568 | -0.342 | -0.5444 | No |
| 41 | HMGB3 |  |  | 577 | -0.348 | -0.5517 | No |
| 42 | CACYBP |  |  | 589 | -0.352 | -0.5628 | No |
| 43 | DDX39A |  |  | 590 | -0.353 | -0.5596 | No |
| 44 | POC1A |  |  | 611 | -0.363 | -0.5824 | No |
| 45 | G2E3 |  |  | 624 | -0.371 | -0.5947 | No |
| 46 | NEK2 |  |  | 628 | -0.374 | -0.5952 | No |
| 47 | EXOSC8 |  |  | 630 | -0.376 | -0.5931 | No |
| 48 | RNASEH2A |  |  | 633 | -0.378 | -0.5922 | No |
| 49 | MPHOSPH9 |  |  | 644 | -0.384 | -0.6018 | No |
| 50 | GAS2L3 |  |  | 647 | -0.388 | -0.6009 | No |
| 51 | PHF19 |  |  | 648 | -0.391 | -0.5973 | No |
| 52 | TMEM106C |  |  | 654 | -0.397 | -0.6002 | No |
| 53 | SHCBP1 |  |  | 655 | -0.398 | -0.5966 | No |
| 54 | RHNO1 |  |  | 664 | -0.406 | -0.6033 | No |
| 55 | SAE1 |  |  | 668 | -0.408 | -0.6035 | No |
| 56 | KIF18A |  |  | 673 | -0.412 | -0.6050 | No |
| 57 | NUDT1 |  |  | 674 | -0.413 | -0.6013 | No |
| 58 | RBMX |  |  | 682 | -0.421 | -0.6066 | No |
| 59 | H2AFV |  |  | 684 | -0.423 | -0.6040 | No |
| 60 | CKAP5 |  |  | 694 | -0.426 | -0.6119 | No |
| 61 | SLC25A5 |  |  | 695 | -0.426 | -0.6080 | No |
| 62 | DIAPH3 |  |  | 696 | -0.427 | -0.6041 | No |
| 63 | RANBP1 |  |  | 697 | -0.427 | -0.6003 | No |
| 64 | ANP32B |  |  | 699 | -0.431 | -0.5977 | No |
| 65 | PARPBP |  |  | 705 | -0.434 | -0.6002 | No |
| 66 | MZT1 |  |  | 711 | -0.437 | -0.6028 | No |
| 67 | TTK |  |  | 724 | -0.454 | -0.6143 | No |
| 68 | RAD21 |  |  | 728 | -0.458 | -0.6141 | No |
| 69 | SPAG5 |  |  | 729 | -0.458 | -0.6099 | No |
| 70 | LBR |  |  | 739 | -0.463 | -0.6174 | No |
| 71 | NUCKS1 |  |  | 744 | -0.471 | -0.6184 | Yes |
| 72 | PTMA |  |  | 745 | -0.475 | -0.6140 | Yes |
| 73 | TK1 |  |  | 747 | -0.480 | -0.6110 | Yes |
| 74 | MIS18A |  |  | 750 | -0.483 | -0.6092 | Yes |
| 75 | KNSTRN |  |  | 751 | -0.485 | -0.6048 | Yes |
| 76 | PRR11 |  |  | 755 | -0.488 | -0.6043 | Yes |
| 77 | SPDL1 |  |  | 758 | -0.491 | -0.6024 | Yes |
| 78 | CDCA3 |  |  | 759 | -0.497 | -0.5979 | Yes |
| 79 | TRIP13 |  |  | 760 | -0.498 | -0.5934 | Yes |
| 80 | DCP2 |  |  | 762 | -0.500 | -0.5901 | Yes |
| 81 | MXD3 |  |  | 763 | -0.502 | -0.5856 | Yes |
| 82 | GMNN |  |  | 766 | -0.510 | -0.5835 | Yes |
| 83 | RTKN2 |  |  | 767 | -0.511 | -0.5789 | Yes |
| 84 | LRR1 |  |  | 768 | -0.512 | -0.5742 | Yes |
| 85 | KIF5B |  |  | 770 | -0.515 | -0.5709 | Yes |
| 86 | CMC2 |  |  | 771 | -0.518 | -0.5662 | Yes |
| 87 | BTG1 |  |  | 772 | -0.520 | -0.5614 | Yes |
| 88 | DTYMK |  |  | 774 | -0.522 | -0.5580 | Yes |
| 89 | STMN1 |  |  | 778 | -0.529 | -0.5571 | Yes |
| 90 | DEPDC1B |  |  | 779 | -0.533 | -0.5523 | Yes |
| 91 | CENPA |  |  | 782 | -0.538 | -0.5500 | Yes |
| 92 | SKA3 |  |  | 783 | -0.539 | -0.5451 | Yes |
| 93 | NCAPD2 |  |  | 784 | -0.540 | -0.5402 | Yes |
| 94 | RPA3 |  |  | 788 | -0.550 | -0.5391 | Yes |
| 95 | CDC20 |  |  | 793 | -0.562 | -0.5392 | Yes |
| 96 | HMGN2 |  |  | 795 | -0.564 | -0.5354 | Yes |
| 97 | RACGAP1 |  |  | 796 | -0.566 | -0.5302 | Yes |
| 98 | HMGB1 |  |  | 797 | -0.567 | -0.5251 | Yes |
| 99 | HES6 |  |  | 800 | -0.569 | -0.5225 | Yes |
| 100 | TUBB |  |  | 802 | -0.570 | -0.5186 | Yes |
| 101 | TUBB4B |  |  | 804 | -0.573 | -0.5147 | Yes |
| 102 | CENPN |  |  | 805 | -0.574 | -0.5095 | Yes |
| 103 | CDCA8 |  |  | 808 | -0.585 | -0.5068 | Yes |
| 104 | PLK1 |  |  | 809 | -0.586 | -0.5015 | Yes |
| 105 | DEK |  |  | 812 | -0.591 | -0.4987 | Yes |
| 106 | FOXM1 |  |  | 814 | -0.593 | -0.4946 | Yes |
| 107 | DEPDC1 |  |  | 815 | -0.596 | -0.4892 | Yes |
| 108 | CDCA2 |  |  | 816 | -0.597 | -0.4838 | Yes |
| 109 | LMNB1 |  |  | 818 | -0.606 | -0.4796 | Yes |
| 110 | PIF1 |  |  | 822 | -0.616 | -0.4779 | Yes |
| 111 | AURKA |  |  | 824 | -0.623 | -0.4735 | Yes |
| 112 | CKAP2L |  |  | 826 | -0.625 | -0.4692 | Yes |
| 113 | CCDC34 |  |  | 827 | -0.626 | -0.4635 | Yes |
| 114 | AURKB |  |  | 829 | -0.634 | -0.4590 | Yes |
| 115 | BUB3 |  |  | 834 | -0.641 | -0.4584 | Yes |
| 116 | CENPW |  |  | 835 | -0.643 | -0.4526 | Yes |
| 117 | CDKN3 |  |  | 836 | -0.653 | -0.4466 | Yes |
| 118 | BUB1 |  |  | 837 | -0.653 | -0.4407 | Yes |
| 119 | ANP32E |  |  | 841 | -0.676 | -0.4385 | Yes |
| 120 | KIF22 |  |  | 842 | -0.678 | -0.4323 | Yes |
| 121 | BIRC5 |  |  | 843 | -0.679 | -0.4261 | Yes |
| 122 | CENPM |  |  | 844 | -0.682 | -0.4199 | Yes |
| 123 | ZWINT |  |  | 847 | -0.692 | -0.4163 | Yes |
| 124 | SPC24 |  |  | 848 | -0.694 | -0.4099 | Yes |
| 125 | CENPH |  |  | 849 | -0.694 | -0.4036 | Yes |
| 126 | ECT2 |  |  | 854 | -0.717 | -0.4023 | Yes |
| 127 | KIF2C |  |  | 855 | -0.721 | -0.3958 | Yes |
| 128 | NUF2 |  |  | 856 | -0.721 | -0.3892 | Yes |
| 129 | CENPK |  |  | 857 | -0.722 | -0.3827 | Yes |
| 130 | ANLN |  |  | 860 | -0.733 | -0.3786 | Yes |
| 131 | KIF4A |  |  | 861 | -0.736 | -0.3719 | Yes |
| 132 | HMMR |  |  | 863 | -0.740 | -0.3665 | Yes |
| 133 | FBXO5 |  |  | 864 | -0.742 | -0.3598 | Yes |
| 134 | CCNB2 |  |  | 865 | -0.745 | -0.3530 | Yes |
| 135 | ARHGAP11A |  |  | 867 | -0.756 | -0.3474 | Yes |
| 136 | NDC80 |  |  | 869 | -0.762 | -0.3418 | Yes |
| 137 | BUB1B |  |  | 871 | -0.766 | -0.3361 | Yes |
| 138 | CCNA2 |  |  | 872 | -0.770 | -0.3292 | Yes |
| 139 | KIF23 |  |  | 873 | -0.772 | -0.3221 | Yes |
| 140 | MELK |  |  | 874 | -0.776 | -0.3151 | Yes |
| 141 | TMPO |  |  | 875 | -0.778 | -0.3080 | Yes |
| 142 | KIF20B |  |  | 876 | -0.779 | -0.3009 | Yes |
| 143 | CEP55 |  |  | 877 | -0.780 | -0.2938 | Yes |
| 144 | CKAP2 |  |  | 878 | -0.781 | -0.2867 | Yes |
| 145 | PTTG1 |  |  | 880 | -0.791 | -0.2809 | Yes |
| 146 | H2AFX |  |  | 882 | -0.799 | -0.2749 | Yes |
| 147 | KIFC1 |  |  | 883 | -0.807 | -0.2676 | Yes |
| 148 | KIF14 |  |  | 885 | -0.809 | -0.2615 | Yes |
| 149 | UBE2S |  |  | 887 | -0.815 | -0.2554 | Yes |
| 150 | H2AFZ |  |  | 893 | -0.849 | -0.2542 | Yes |
| 151 | KPNA2 |  |  | 895 | -0.858 | -0.2477 | Yes |
| 152 | DBF4 |  |  | 896 | -0.864 | -0.2399 | Yes |
| 153 | SMC2 |  |  | 897 | -0.867 | -0.2320 | Yes |
| 154 | CENPE |  |  | 899 | -0.873 | -0.2254 | Yes |
| 155 | PBK |  |  | 900 | -0.880 | -0.2174 | Yes |
| 156 | SMC4 |  |  | 902 | -0.886 | -0.2106 | Yes |
| 157 | MIS18BP1 |  |  | 904 | -0.889 | -0.2038 | Yes |
| 158 | CCNB1 |  |  | 906 | -0.895 | -0.1970 | Yes |
| 159 | CKS1B |  |  | 909 | -0.912 | -0.1913 | Yes |
| 160 | HJURP |  |  | 910 | -0.921 | -0.1830 | Yes |
| 161 | CKS2 |  |  | 911 | -0.922 | -0.1746 | Yes |
| 162 | GTSE1 |  |  | 912 | -0.941 | -0.1660 | Yes |
| 163 | KIF11 |  |  | 913 | -0.957 | -0.1573 | Yes |
| 164 | PRC1 |  |  | 919 | -0.980 | -0.1549 | Yes |
| 165 | MAD2L1 |  |  | 921 | -0.990 | -0.1473 | Yes |
| 166 | UBE2T |  |  | 922 | -0.994 | -0.1382 | Yes |
| 167 | DLGAP5 |  |  | 923 | -0.998 | -0.1291 | Yes |
| 168 | SPC25 |  |  | 924 | -1.033 | -0.1198 | Yes |
| 169 | ESCO2 |  |  | 926 | -1.067 | -0.1114 | Yes |
| 170 | UBE2C |  |  | 928 | -1.108 | -0.1026 | Yes |
| 171 | NUSAP1 |  |  | 929 | -1.112 | -0.0925 | Yes |
| 172 | TPX2 |  |  | 930 | -1.124 | -0.0823 | Yes |
| 173 | NCAPG |  |  | 931 | -1.142 | -0.0719 | Yes |
| 174 | ASPM |  |  | 932 | -1.146 | -0.0615 | Yes |
| 175 | CENPF |  |  | 935 | -1.200 | -0.0532 | Yes |
| 176 | HMGB2 |  |  | 937 | -1.238 | -0.0432 | Yes |
| 177 | CDK1 |  |  | 938 | -1.248 | -0.0319 | Yes |
| 178 | RRM2 |  |  | 940 | -1.403 | -0.0205 | Yes |
| 179 | MKI67 |  |  | 941 | -1.407 | -0.0077 | Yes |
| 180 | TOP2A |  |  | 942 | -1.417 | 0.0052 | Yes |
Table: GSEA details [plain text format]

  

Fig 2: FELDMAN\_2020\_G2-M: Random ES distribution      
 Gene set null distribution of ES for **FELDMAN\_2020\_G2-M**
